# Long-Term Health Consequences of SARS-CoV-2: Assumptions Based on SARS-CoV-1 and MERS-CoV Infections

**DOI:** 10.3390/diagnostics12081852

**Published:** 2022-07-31

**Authors:** Ashutosh Khaswal, Vivek Kumar, Subodh Kumar

**Affiliations:** 1 Department of Biotechnology, IMS Engineering College, NH-24, Ghaziabad 201009, Uttar Pradesh, India; khaswalashu@gmail.com (A.K.); vivek.ucms@gmail.com (V.K.); 2 Center of Emphasis in Neuroscience, Department of Molecular and Translational Medicine, Paul L. Foster School of Medicine, Texas Tech University Health Sciences Center, 5001 El Paso Drive, El Paso, TX 79905, USA

**Keywords:** SARS-CoV-1, MERS-CoV, SARS-CoV-2, manifestations, health consequences

## Abstract

Coronavirus Disease-2019 (COVID-19) is one of the worst pandemics in the history of the world. It is the third coronavirus disease that has afflicted humans in a short span of time. The world appears to be recovering from the grasp of this deadly pandemic; still, its post-disease health effects are not clearly understood. It is evident that the vast majority of COVID-19 patients usually recovered over time; however, disease manifestation is reported to still exist in some patients even after complete recovery. The disease is known to have left irreversible damage(s) among some patients and these damages are expected to cause mild or severe degrees of health effects. Apart from the apparent damage to the lungs caused by SARS-CoV-1, MERS-CoV, and SARS-CoV-2 infection, COVID-19-surviving patients display a wide spectrum of dysfunctions in different organ systems that is similar to what occurs with SARS-CoV-1 and MERS diseases. The major long COVID-19 manifestations include the following aspects: (1) central nervous system, (2) cardiovascular, (3) pulmonary, (4) gastrointestinal, (5) hematologic, (6) renal and (7) psycho-social systems. COVID-19 has a disease display manifestation in these organs and its related systems amongst a large number of recovered cases. Our study highlights the expected bodily consequences of the pandemic caused by SARS-CoV-2 infection based on the understanding of the long-term effects of SARS-CoV-1 and MERS-CoV.

## 1. Introduction

The COVID-19 disease was declared a pandemic by WHO at the beginning of 2020; since then, it has led to widespread health, social and economic damages worldwide. COVID-19 is an illness caused by the virus known as severe acute respiratory syndrome coronavirus 2 (SARS-CoV-2). COVID-19 is an enveloped, positive single-stranded RNA virus that not only infects humans, but also a wide range of animals [1,2]. The virus enters the host cells via the binding of its spike proteins (made up of glycoproteins) to the human cell-surface receptor ACE2 (angiotensin-converting enzyme 2), abundantly present in the epithelial cells of the nasal cavity and alveoli [3]. A study from Johns Hopkins University found that case fatality rates, i.e., percentage of morbidity amongst confirmed COVID-19 patients, varies between 1–7%. This variation significantly relies on one or a combination of several established factors, such as testing efficacy, population ages and local pandemic response policies [2,4]. For instance, case fatality rates in Italy are <3% in the population younger than 60 years of age and more than 30% fatality in the groups aged 80 years or above [5]. However, with rapid vaccination and the development of herd immunity worldwide, the pandemic is rapidly shrinking in the world. Thus, most of the world population has recovered from COVID-19, while some patients, i.e., infected from COVID-19, are likely experiencing long-term after-effects of the SARS-CoV-2 over major systems of the human body, including the central nervous system, pulmonary, hematologic, cardiovascular, gastrointestinal and renal systems.

Various human coronaviruses (CoV) have been reported to date that can cause mild illness in the human body, such as fever, headache, gastrointestinal infections and common cold. Two species of coronaviruses, i.e., severe acute respiratory syndrome coronavirus (SARS-CoV) and Middle East respiratory syndrome coronavirus (MERS-CoV), have resulted in epidemics around the world by crossing the species barrier and leading to high mortality and pathogenicity in humans [6,7]. Both viruses are highly pathogenic in nature, having caused a fatality rate of 9.6% (2003–2004) and 34.3% (2012–present) [7,8]. Both SARS-CoV-1 and MERS-CoV cause post-recovery health complications in the human body, and most of these health consequences are present in SARS-CoV-2-recovered patients. 

SARS-CoV-2 is the third virus of the coronavirus family that has affected humans. The purpose of our article is to highlight the expected bodily consequences of the pandemic caused by SARS-CoV-2 infection based on the understanding of the long-term effects of SARS-CoV-1 and MERS-CoV. We also discuss the health outcomes of patients recovered from COVID-19.

## 2. Taxonomy of Coronaviruses

Coronaviruses are classified as a single-strand RNA viruses with an envelope that has the capability of causing infection in a wide range of hosts, including animals, humans and other mammalian species [6]. CoVs belong to the *Coronaviridae* family of the *Nidovirales* order. The taxonomical features of CoVs have the following hierarchy: Category—Coronaviruses; Realm—*Riboviria*; Order—*nedovirales*; Suborder—*cornidovirineae*; Family—*coronaviridae*; Subfamily—*orthocoronaviridae*; Genus—*betacoronovirus*; Lineage—B: Species—severe acute respiratory syndrome-related coronavirus (SARS-CoV-2, SARS-CoV-Urbani, SARS-CoVGZ-02, Bat SARS-CoV-1, Civet SARSCoVSZ3/2003, SARS-CoVPC4-227, SARSr-COVBtKY72, SARSr-CoVRatG13); Lineage—C: Species—Middle east respiratory syndrome (MERS) [9,10]. CoVs are well known for their ability to adapt to the environment, alter tissue tropism, cross the species barrier and mutate rapidly to different epidemiological situations [11]. In this review paper, SARS-CoV-1, MERS and SARS-CoV-2 are studied to follow the long-term consequences of these lineage B species of coronaviruses. Table 1 represents the comparative analysis of CoVs that cause differences in the epidemic scale and the severity of the consequences [9,10,11,12,13,14,15].

## 3. SARS-CoV-2 Virus

**Figure 1:**
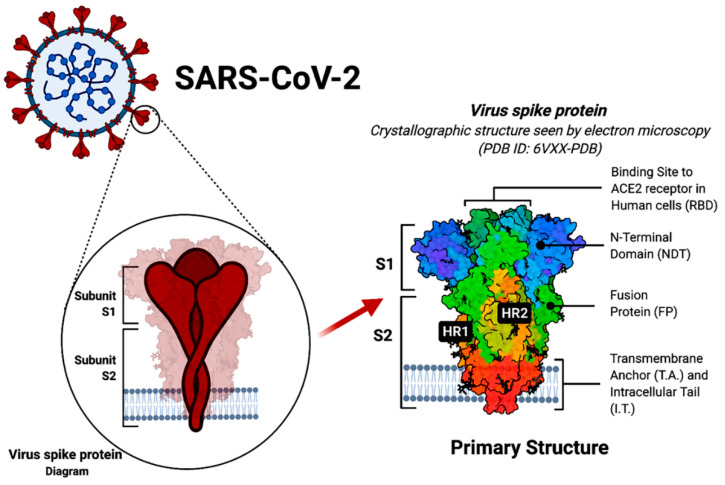
A typical SARS-CoV-2 virus and spike proteins. Detailed crystallographic structure of the protein including the ACE2 receptor-binding site in human cells, (HR1 and HR2—heptad repeat 1 and 2).

SARS-CoV-2 contains the largest viral genome among all the RNA viruses, ranging from 27 to 32 kb. Receptor-mediated endocytosis is the main process of virus entry into host cells. The human ACE2 receptor is the main SARS-CoV-2 spike-binding receptor, known to express on kidney, blood vessels, heart cells and, most commonly, in the respiratory tract system [16]. The acuteness of the SARS-CoV-2 pandemic and its rapid spread all over the world mobilized an unprecedented effort of scientific communities, such as those of the fields of medicine, biology, health, bioinformatics and computer science, leading to the rapid development of several vaccines [17,18,19,20]. Reddy et al. observed that SARS-CoV-2 controls mitochondria indirectly when it enters the host (human body), resulting in manipulating or regulating mitochondrial functions just by changing open reading frames, such as ORF-9B [21,22]. Once the virus controls the mitochondrial function of a cell, it impairs the immune function of host cells and promote viral replication, causing COVID-19 disease [23]. Figure 1 represents the in-depth look into the structure of the SARS-CoV-2 spike glycoprotein (created with biorender.com, 15 June 2022).

**Figure 2:**
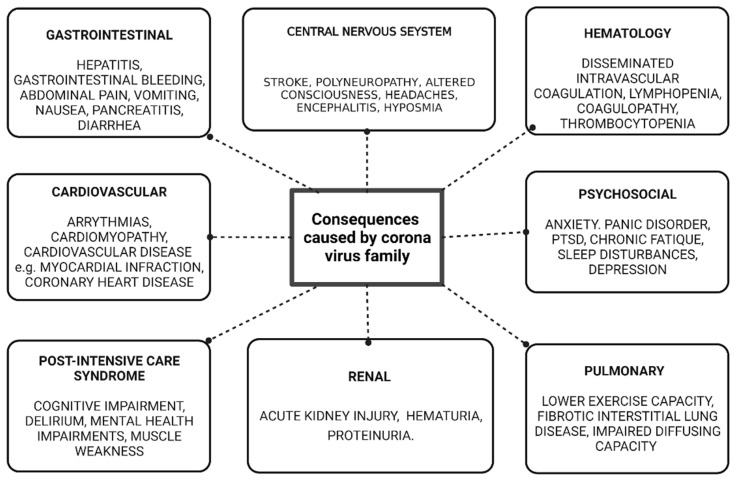
Common long-term health effects of COVID-19 in recovered patients.

The focus of this review is to access the possible long-term complications of COVID-19 in the recovered patients based on the assumptions from SARS-1 and MERS infections. The most common long-term consequences that a patient can experience after SARS-CoV-2 infection are presented in Figure 2, which include CNS manifestations, hypercoagulability, pulmonary manifestations, cardiovascular manifestations, renal manifestations, gastrointestinal manifestations and psycho-social manifestations.

The majority of health complications amongst surviving patients stem from the prothrombotic state. Thrombosis was amongst the major causes of mortality in severe COVID-19 patients. Moreover, thrombosis prevention by the use of various anti-clotting medications, i.e., low-molecular-weight heparin, was one of the main lines of treatment since the onset of pandemic. Several hypotheses explain the molecular mechanism behind the hypercoagulable state due to thrombosis that has been seen among the majority of COVID-19 patients. As thrombosis is a major root of most of the severe complications in COVID-19 patients, including brain hemorrhage, heart attacks and vital organ failures, it needs extensive research by medical researchers and scientists of various other related fields to control and overcome health outcomes associated with the hypercoagulability state in recovered cases [24].

### 3.1. COVID-19-Induced Thrombosis: Root of the Major Complications Amongst Recovered Patients

A hypercoagulable state is a major consequence of SARS-CoV-2 virus infection. It is believed that some patients stay in a pro-coagulative state even after recovery. The virus infection leads to either multiple site clotting events or hemorrhage. Thrombosis prevention is one of the main lines of treatment and prevention of deaths amongst critically ill COVID-19 patients [24,25]. A group of 22 Chinese researchers reported that 4889 confirmed COVID-19 patients showed a high prevalence of coagulopathy with elevated D-dimer levels and prolonged prothrombin time (PT) in more severe patients [26]. Therefore, it is evident that thrombotic complications are emerging as a significant cause of morbidity amongst COVID-19 recovered patients and need extensive research. Many studies explain that the molecular mechanism behind the pro-coagulant state seen among COVID-19 patients includes different receptor bindings, cytokine storm and direct viral endothelial damage [4,27]. It has also been reported that SARS-CoV-2 binds to CLEC4M receptor (a receptor that participates in the clearance of Factor VIII and Von Willebrand Factor) [27,28]. The competitive binding of SARS-CoV-2 to CLEC4M results in a decreased clearance of Factor VIII and Von Willebrand Factor, which promotes a pro-coagulative state in the human body after recovery [29,30].

Hypercoagulability has another mechanism that has been described in severe cases as staging a “cytokine storm” in the system, brought about by the rapid elevation of pro-inflammatory cytokines (GM-CSF and IL-6) through SARS-CoV-2-mediated Th1-cell activation [31]. Consequently, GM-CSF activates inflammatory monocytes (CD14+ and CD16+) to produce even large quantities of IL-6, tumor necrosis factor-α (TNF-α) and IL-1 [32,33]. These well-known cytokines were found to be at high levels in patients with sepsis associated with a hypercoagulable status, as seen in disseminated intravascular coagulation (DIC) [34]. However, in COVID-19 patients, IL-6 seems to be the key mediator in initiating hypercoagulation. Folman et al. also found that IL-6 induces the expression of tissue factor (TF) in inflamed tissues and stimulates megakaryopoiesis [35]. An increase in TF expression could be seen in patients with COVID-19 due to damage and inflammation of the lung tissue that leads to the increase in IL-6 levels [36,37]. It has been observed that IL-6 stimulates the production of fibrinogen and Factor VIII. Fibrinogen and Factor VIII activate the endothelial cells to induce vascular permeability by stimulating vascular endothelial growth factor (VEGF) secretion, which ultimately causes blood clotting [36].

**Figure 3:**
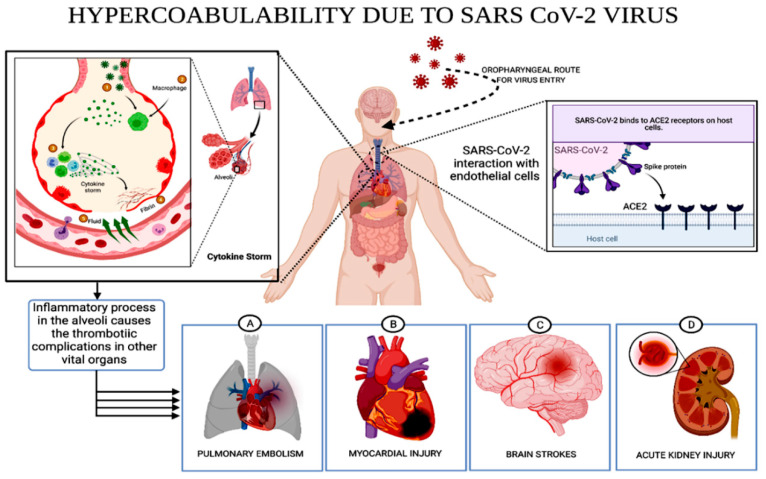
Entry of SARS-CoV-2 into the body through the respiratory system. The virus induces thrombosis in the lungs (mechanism of cytokine storm is also represented) and, through circulatory system clots, the virus reaches the various body system and leads to complications in human organs, such as the lungs, heart, brain and kidneys (created with biorender.com, 22 June 2022).

We also critically discussed the mechanism of cytokine storm in five major steps: (1) coronavirus infects lungs cells; (2) immune cells, including macrophages, identify the virus and produce cytokines; (3) cytokines attract more immune cells, such as white blood cells, which in turn produce more cytokines, creating a cycle of inflammation that damages lung cells; (4) damage can occur through the formation of fibrin; (5) weakened blood vessels allow fluid to seep in and fill the lung cavities, leading to respiratory failure, whereas the A, B, C and D sites represent the expected body systems that can experience major complications due to hypercoagulability and cytokine storm (Figure 3).

### 3.2. Expected Long-Term Thrombotic Health Effects

During the recovery period of COVID-19 patients, thrombotic events reach the various parts of the body through the circulatory system. Blood clots are likely to cause various micro- and macro-damages in different organs that could be the significant reason for the development of various disorders in COVID-19-infected patients. Scientists noticed that thrombotic complications may arise in surviving COVID-19 patients due to hypercoagulability, including pulmonary embolism, myocardial injury, brain stroke, deep vein thrombosis and other inflammatory responses [24,38].

## 4. Central Nervous System Manifestations

COVID-19 also affects the central nervous system (CNS) functions. The worldwide incidence of neurological and psychiatric disorders has arisen alarmingly. According to the recently published studies, one-third of the recovered patients experience neurological disorders within six months of recovery [39]. A group of scientists reported that COVID-19-causative viral particles penetrate into the CNS via cribriform plate, systemic circulation, or olfactory bulb to access the brain by damaging the capillary endothelium, which results in CNS manifestations [40].

Accumulating evidence indicates strong changes in the neurobiology of COVID-19-affected patients. COVID-19-induced cytokine storm is recognized as the main mediator for CNS consequences. This may affect the CNS primarily in two different ways: (1) cytokine-storm-induced hypercoagulable state via induced arterial occlusion or venous thrombosis that increases the susceptibility of an individual to strokes; (2) massive release of pro-inflammatory cytokines, including TNF-α crosses the blood–brain barrier. TNF-α and other cytokines activate microglia and astrocytes. These cells not only phagocytose damaged cells, but also release mediators of inflammation, including glutamate, quinolinic acid, ILs and complement proteins. N-methyl-D-aspartate and glutamate upregulation is induced by quinolinic acid. This possibly induces a variety of CNS manifestations, including altered memory [41,42,43,44].

**Figure 4:**
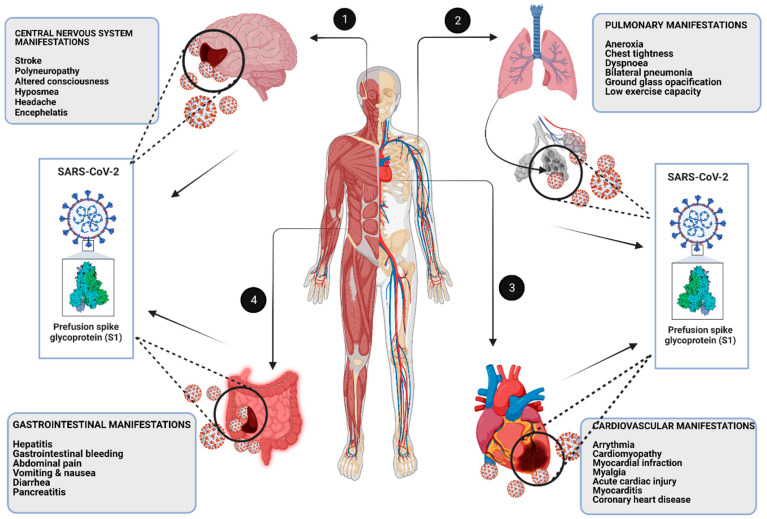
The major long-term consequences on vital organs of human body due to COVID-19: (1) central nervous system manifestations, (2) pulmonary manifestations, (3) cardiovascular manifestations and (4) gastrointestinal manifestations.

SARS-CoV-2 is believed to follow four different types of mechanisms that directly contribute towards CNS manifestations: (1) cerebrovascular dysfunction, (2) systemic inflammation, (3) direct viral encephalitis and (4) peripheral organ dysfunction (Figure 4). Long-term CNS manifestations may arise by any of these mechanisms [45,46,47]. Helms et al. (2020) summarized motor deficiency and cognitive impairments amongst one-third of patients discharged from hospital after recovery from COVID-19 [48]. Neurological disorders and cognitive decline are promoted by systemic inflammation; it strongly suggests that patients suffering from SARS-CoV-2 will likely experience long-term CNS manifestations that can further increase the risk of Alzheimer’s disease and many more neuropathological disorders. SARS-CoV-2 has potential implications in Alzheimer’s disease progression. Neuroinflammation caused by SARS-CoV-2 plays a critical role in AD pathology. Immune hyperactivation and excessive inflammation also contribute to the neurodegeneration in COVID-19-recovered patients and elderly individuals are more susceptible to neurological disorders and cognitive impairment [49].

The percentage of patients affected with different types of CNS manifestations after COVID-19 are summarized in Table 2.

Middle East respiratory syndrome coronavirus (MERS-CoV) and severe acute respiratory syndrome-1 (SARS-CoV-1) have been noticed to be linked with neurological disorders, including stroke [50], encephalitis [51] and polyneuropathy [52,53]. Similarly, in a comparison of these central nervous system manifestations with SARS-CoV-2, Paterson et al. (2020) reviewed the radiological, neuropathological, clinical and laboratory data of 43 COVID-19 patients and found that majority suffered from CNS manifestations, such as ischemic stroke, inflammatory CNS syndrome, transient encephalopathies and peripheral neurological disorders [39]. SARS-CoV-2 has been reported to be linked with stroke [54,55], encephalitis [39] and polyneuropathy [56], along with common CNS disorders, such as weakness, hyposomnia, altered consciousness, headache, mood swings and brain fog [55,57]. Keeping this in mind, all the above-mentioned observations and early onset of a wide array of geriatric–neurological disorders can be anticipated among healthy and much younger population. Other related disorders may affect the cognitive and working efficiency amongst this group of people.

### Expected Long-Term CNS Health Effects

Neurological disorders are one of the most frequently reported disorders amongst recovered patients. Any kind of functional variability at the neurological level may have cascading effects on one or more systems of the body. Although the reversibility of damages left in the brain by infection is difficult to predict, by looking at the current morbidities among patients, it can be said that the early onset of a wide array of geriatric–neurological disorders could be anticipated. Moreover, chronic disorders such as hyposomnia and weakness are expected to gradually affect and weaken the immune system, making them more susceptible to other illnesses, such as Guillain–Barre syndrome and Bell’s palsy [56,58]. Age-related neuro-impairment might also have an effect on cognitive ability that, in turn, is likely to diminish the individual’s working efficiency with time. A recent study (July 2022) from Bangalore, India reported a sharp spike in of nearly 25% in insomnia, anxiety and memory loss after a long time of recovery from COVID-19. This study also found that the health of patients with pre-existing neurological conditions, such as Parkinson’s or Alzheimer’s diseases, worsen after COVID-19 [58,59]. More details about CNS manifestations are summarized in Table 2.

**Table 2:** The most common CNS manifestations noticed amongst COVID-19-recovered patients.

S.No	Total No. of Patients	Mean Age (Years)	COVID-19 Status	CNS Manifestations	Total % of Patients Experience CNS Manifestations	References
1	43 patients Gender—24 males and 19 females	16-85	29 patients were SARS-CoV-2 PCR-positive	Encephalopathies	23.2%	[39]
				Inflammatory CNS syndromes, including encephalitis, acute disseminated encephalomyelitis (ADEM) and myelitis	28%	
				Ischemic strokes	18.6%	
				Peripheral syndrome	18.6%	
2	64 patients	63	Acute respiratory distress syndrome caused by COVID-19	Agitation	69%	[48]
				Confusion	65%	
				Signs of corticospinal tract dysfunction	67%	
				Cerebral ischemic stroke	23%	
				Dysexecutive syndrome	36%	
3	214 patients Gender—87 males (40.7%) and 127 females (59.3%)	58.2	88 patients with severe COVID-19 126 patients with non-severe COVID-19	Dizziness	17%	[53]
				Headache	13%	
				Impaired level of consciousness	8%	
				Acute stroke	3%	
				Ataxia	<1.0%	
				Seizures	<1.0%	
4	235 patients	63	168 intubated patients suffered from severe COVID-19	Neurological symptoms	22%	[60]

## 5. Pulmonary Manifestations

Pulmonary system is one of the top sites for COVID infection. All human-infecting coronaviruses have common genetic similarities; the main one is that the lungs are the primary site of infection for all these viruses (SARS-CoV-1, MERS-CoV and SARS-CoV-2). They all lead to acute respiratory distress syndromes [61]. A certain degree of health outcomes in long-term COVID-19 cases can be predicted from SARS-CoV-1 and MERS-CoV epidemics. Several investigations suggested that SARS-CoV-1-infected patients had a reduced carbon monoxide diffusion capacity and reduced exercise capacity in the body due to impairment of the intra-alveolar diffusion pathway after a duration of about one-half to fifteen years of infection [62,63]. A group of scientists analyzed 110 SARS-CoV-1-infected patients and noticed that patients experienced minor abnormalities in their chest radiography, which showed lower exercise capacity as compared to a normal person after 6 months of symptoms onset [62,63]. A 2-year follow-up study reported that 52% of SARS-CoV-1-infected patients had impairments in persistent diffusing capacity and reduced exercise capacity [64]. In a 15-year follow-up study of 71 patients who recovered from SARS-CoV-1, the greatest extent of recovery was experienced from pulmonary interstitial abnormalities followed by the subsequent functional decline within the first years after infection. However, Hui et al. (2005) reported that 4.6% of SARS-CoV-1 patients had interstitial lung abnormalities even after 15 years [65]. Figure 4 represents the major pulmonary manifestations in recovered COVID-19 patients.

Similar long-term abnormalities have been reported for MERS-CoV, with 36% of patients who recovered from MERS-CoV experiencing abnormal chest radiographs, including lung fibrosis, ground-glass opacity and pleural swelling [66]. Virus-induced immunopathological events are thought to contribute to the pulmonary manifestations caused by SARS-CoV-1 and MERS-CoV [67]. Specifically, these may include rapid virus replication that leads to greater cytopathic effects, predominant infection of alveolar epithelial cells (i.e., type I and II pneumocytes) and increased production of pro-inflammatory cytokines and chemokines, which in turn recruit fibroblasts and induce their differentiation into myofibroblasts [68,69]. Furthermore, the ability of SARS-CoV-1 and MERS-CoV antagonists subsequently delays interferon responses and dysregulates the inflammatory response [68].

In relation to COVID-19, a meta-analysis of 31 articles and approximately 50,000 patients with SARS-CoV-2 reported that 29% of patients developed acute respiratory distress syndrome, 76% had double pneumonia, 20% had unilateral pneumonia and 31% reported chest distress [70] (Table 3). A study of 138 hospitalized patients of COVID-19 exhibited bilateral involvement of chest computed tomography scan, which showed pulmonary abnormalities, including ground-glass opacities (70%), irregular lesions (54%) and broncho vascular bundle thickening (40%) [71]. A total of 81 patients were studied who had SARS-CoV-2 pneumonia. The asymptomatic COVID-19 patients scan showed chest abnormalities as confirmed by computed tomography imaging [72]. These abnormalities were found to be rapidly evolved from focal unilateral to diffuse bilateral ground-glass opacities [72].

Patients who have recovered from COVID-19 may develop irreversible fibrotic interstitial lung disease due to the persistence of chronic inflammation [73], although pulmonary function abnormalities are assumed to be reversible with time or treatments. Patients with severe COVID-19 exhibit excessive inflammatory damage due to a failed anti-inflammatory response and, subsequently, excessive proinflammatory cytokines that damage epithelial and endothelial cells of the lungs [74]. Importantly, 54% of asymptomatic positive cases from the cruise ship Diamond Princess had lung opacities on computed tomography [75], with a similar prevalence reported in asymptomatic or minimally symptomatic patients in Italy [76]. However, without prospective studies, the long-term effect(s) of COVID-19 infection on the lungs cannot be determined.

### Expected Long-Term Pulmonary Health Effects

There are contrasting similarities amongst the symptoms produced by the different human-infecting coronaviruses. It can be speculated that the patients recovered from COVID-19 are likely to exhibit symptoms and disorders similar to those of MERS-CoV and SARS-CoV-1. Therefore, a similar degree of ailments comparable to these two diseases can be anticipated amongst COVID-19 recovered patients. However, the individual susceptibility is hard to define, but it is likely that the risk of developing above-mentioned disorders is influenced by various factors, such as the extent of irreversible damage to lungs, age, gender, environmental factors such as physical activity, diet and other unknown factors, as well as the majority of COVID-19-associated long-term illness cases will have a respiratory distress of different degree. It is highly likely that respiratory stressors, such as pollution, heavy work and low air humidity, can further aggravate respiratory complications. The weakening of the lungs due to COVID-19 infection(s) will make patients susceptible to infectious diseases; a recent report from WHO has attributed a significant rise in the deaths due to tuberculosis in more than a decade due to the pandemic [59,77].

**Table 3:** Major pulmonary manifestations noticed as a consequence of SARS-CoV-2 infection (* N/A= not known).

S.No.	Total No. of Patients	Mean Age (Years)	COVID-19 Status	Pulmonary Manifestations	Total % of Patients Experience Pulmonary Manifestations	Reference
1	46,959 patients’ meta-analysis	N/A	COVID-19 infection impacted patients	Fever	87.3%	[70]
				Cough	58.1%	
				Dyspnea	38.3%	
				Muscle soreness or fatigue	35.5%	
				Chest distress	31.2%	
				Bilateral pneumonia	75.7%	
				Ground-glass opacification	69.9%	
2	81 patients Gender—52% men; 48% women	49.5	COVID-19 infection impacted patients	Anorexia	22%	[72]
				Chest tightness	59%	
				Cough	19%	
				Sputum	26%	
				Rhinorrhea	1%	
				Dyspnea	42%	
3	55 Patients	N/A	4 mild, 47 moderate and 4 severe COVID-19 infection	Radiologic abnormalities consistent with pulmonary dysfunction, such as interstitial thickening and evidence of fibrosis	71%	[78]
				Persistent symptoms of pulmonary disorder	64%	
				Decreased carbon monoxide diffusion capacity	25%	
4	57 patients	N/A	40 non-severe cases and 17 severe cases of COVID-19 infection	Forced vital capacity (FVC <80%)	10.5%	[79]
				Forced expiratory volume (FEV1 <80%)	8.7%	
				(FEV1/FVC ratio <80%)	43.8%	
				Total lung capacity (TLC <80%)	12.3%	
				Diffusing capacity of lung for carbon monoxide (DLCO <80%)	52.6%	
5	139 patients Gender—28% male; 72% female	52	23% (16) hospitalized for COVID-19 infection	Chest pain along with dyspnea and palpitation	42%	[80]

## 6. Cardiopulmonary and Cardiovascular Manifestations

Cardiopulmonary system of heart is responsible for the circulation of oxygenated and deoxygenated blood in the entire body. Due to the infection of the blood circulation in COVID-19 patients, SARS-CoV-2 impacts the whole cardiopulmonary system, not only the heart [66]. Several studies revealed that MERS-CoV, SARS-CoV-1 and pneumonia viruses are linked with cardiopulmonary disorders, including hypotension, cardiomegaly, hypertension, tachycardia and bradycardia [62,70,71]. Recently, Cai and colleagues (2020) conducted a study on 121 patients infected with SARS-CoV-1; those with 25% pre-existing medical illnesses reported cardiovascular manifestations, with 50.4% of patients suffering from hypotension, 14.9% of patients suffering from bradycardia, 71.9% of patients suffering from tachycardia and 10.7% of patients suffering from cardiomegaly [66] (Table 4). However, most of the cardiopulmonary disorders caused by SARS-CoV-1 returned to normal within 3 weeks, except tachycardia, which still perseveres in the patient’s body after three weeks of post recovery [66]. While comparing SARS-CoV-2 with other CoVs of the same family, it has been observed that the patients infected with SARS-CoV-2 virus experienced intense cardio-pulmonary manifestations, such as cardiomegaly, bradycardia, tachycardia and diastolic impairment, which may be largely reversible. However, hyperlipidemia and increased cardiopulmonary disease risk is quite evident years after infection, which can cause long-term cardiopulmonary consequences in COVID-19-infected and in recovered patients [67].

The cardiovascular system is another important part of the body that is severely hit by COVID-19. Hence, the effects of SARS-CoV-2 infection are obvious on the heart and other vascular systems [81]. MERS-CoV, SARS-CoV-1 and pneumonia have been examined by various studies and concluded that these viruses are linked with cardiovascular disorders, including lymphopenia, acute cardiac injury, prolonged prothrombin, arrhythmia, myocardial infraction, myocarditis and myocardial ischemia [62,63]. Desai et al. (2022) reviewed myocarditis as one of the major complications for COVID-19-infected patients that could be a long-term consequence in which the myocytes of the heart muscle cells are destroyed, leading to serious health threats, including heart failure, myopathy or even sudden cardiac death. A group of scientists and doctors had noticed myocarditis cases in COVID-19-infected patients, as summarized in Table 4 [81,82]. Xie et al. (2022) has also noticed the long-term cardiovascular consequences of COVID-19 and provided a comprehensive characterization by reviewing more than 1.5 lacs of patient data from national healthcare databases. They estimated the risks associated with cardiology in SARS-CoV-2-infected patients, including dysrhythmias, ischemic and non-ischemic heart disease, myocarditis, cerebrovascular disorders, pericarditis, thromboembolic disease and heart failure [83]. Researchers observed that patients that suffered from SARS-CoV-1 virus had subclinical diastolic impairment without systolic involvement, which can be reversible after 30 days of recovery period [84]. Other group of scientists revealed that cardiovascular disorder risk could be a long-term consequence of coronavirus by analyzing a 12-year follow-up study reporting health consequences in infected persons who recovered from SARS-CoV-1 viral infection having glucose metabolism disorders (60%), cardiovascular system abnormalities (44%) and persistent hyperlipidemia (69%) [56]. Similarly, when we compare the cardio-vascular manifestations with respect to SARS-CoV-2 virus infection, it has been reported that hypertension and heart disease are among the highest risk factors for COVID-19, partly due to upregulated ACE2 on perivascular pericytes and cardiomyocytes in subjects with these conditions [85]. 

It is also observed that COVID-19 is associated with not only hypertension and heart diseases, but also with other cardiovascular pathologies, including myocarditis [86], cardiomyopathy, myocardial injury and arrhythmias [87,88]. Researchers noticed that SARS-CoV-2 infection amongst young children has striking similarity to Kawasaki disease that has subsequently been named as multisystem inflammatory syndrome in children [MIS-C] [89]. The MIS-C symptoms develops 4–6 weeks after SARS-CoV-2 infection with clinical complications, such as cutaneous manifestations, fever, abdominal pain, diarrhea, organ dysfunction, hyper-inflammatory state and vomiting [90]. By following the fact that cardiovascular disorders along with cardiopulmonary malfunctioning are most notable, including symptomatic myocarditis, coronary artery abnormalities as well as pericarditis, pericardial effusion, vascular regurgitation, hypotension, cardiomegaly, hypertension, tachycardia and bradycardia, it can be suggested that patients that suffered from SARS-CoV-2 will likely experience long-term cardiovascular manifestations that can further increase the susceptibility of CVDs, including myocardial infections and arrhythmias (Figure 4).

**Table 4:** Major cardiovascular manifestations noticed by scientists and researchers in COVID-19 patients.

S.No.	Total No. of Patients	Mean Age (Year)	COVID-19 Status	Cardiovascular and Cardiopulmonary Manifestations	Total % of Patients Experience Cardiovascular Manifestations	Reference
1	139 patients Gender—28% male; 72% female	52	23% (16) hospitalized for COVID-19 infection	Myocarditis	26%	[81]
2	68 patients	N/A	All patients suffered fatal COVID-19 infection	Myocardial damage	7%	[86]
				Myocardial damage with respiratory failure	33%	
3	41 Patients Gender—73% male; 27% female	49	All patients with suspected COVID-19 infection	Cardiovascular disease	15%	[91]
				Myalgia	44%	
				Hemoptysis	5%	
				Dyspnea	55%	
				Lymphopenia	63%	
				Acute cardiac injury	12%	
4	138 Patients. Gender—54.3% male; 45.7% female	22 to 92	All patients suffered severe COVID-19 infection	Lymphopenia	70.3%	[92]
				Acute cardiac injury	7.2%	
				Prolonged prothrombin	58%	
				Arrhythmia	16.7%	
5	68 patients	N/A	All patients with suspected COVID-19 infection	Coronary heart disease	8%	[93]

## 7. Gastro-Intestinal Manifestations

The gastro-intestinal system is another important system of body that is impacted by COVID-19. In the last decade, the majority of the patients that were infected with SARS-CoV-1 virus have reported varieties of digestive-system-related disorders, such as gastrointestinal, pancreatic and hepatic disorders [94,95,96,97]. Similarly, when we compare these gastro-intestinal manifestations with SARS-CoV-2, COVID-19-infected patients experience serious gastro-intestinal disorders, including abdominal pain, diarrhea, anorexia, vomiting, gastro-intestinal bleeding and nausea [32,97] (Figure 4).

Han and colleagues (2020) analyzed 206 COVID-19-infected patients that were admitted to hospital at Wuhan, China, and reported that among 206 patients, 23% had one or more digestive problems, such as vomiting, nausea and diarrhea [98] (Table 5). The remaining suffered from respiratory symptoms or the combination of both gastro-intestinal and respiratory symptoms. The study also suggested that patients with gastro-intestinal symptoms has a longer duration between the onset of symptoms and viral clearance [98]. The presence of SARS-CoV-2 viral particles in fecal matters and the damage of intestinal mucosa strongly supports the potency of COVID-19 virus to exist, infect and replicate in the gastro-intestinal tract [99].

Along with digestive manifestations, hepatic disorders, including liver damage, has been reported during the SARS-CoV-2 pandemic, with increased levels of aspartate amino-transferases and alanine amino-transferases [99]. A group of scientists observed biopsy reports of COVID-19-infected patients and revealed the pathological features of hepatic injuries, including portal inflammation, mild lobular inflammation and moderate microvascular stenosis [100]. Furthermore, pancreatic disorders that have been observed to be caused by SARS-CoV-2 in the human body include increased levels of amylase and lipase along with acute pancreatitis [92,101]. Therefore, digestive symptoms seen in patients show that gastro-intestinal manifestations are emerging as important issues in patients infected with COVID-19. It has been expected as one of the long-term consequences of SARS-CoV-2 infection that could be experienced by patients for many years and needs to be researched.

### Expected Long-Term Gastrointestinal Health Effects

Some of the recovered subjects with one or other GI disorders have experienced long-term morbidity from COVID-19 [102]. Amongst these patients, loss of appetite and liver damages may have major effects on the health of the subjects. It can be anticipated that these patients are likely to suffer from overall health deterioration due to malnutrition. Patients with liver dysfunctions are expected to suffer from various ailments, such as susceptibility to liver infection or even elevated risk of liver cancer.

**Table 5:** Major gastro-intestinal manifestation noticed as a consequence of SARS-CoV-2 infection.

S.No.	Total No. of Patients	Median Age (Year)	COVID-19 Status	Gastro-intestinal Manifestations	Total % of Patients Experience Gastro-Intestinal Manifestations	Reference
1	1099 patients	47	173 patients with severe infection COVID-19 infection	Nausea and vomiting	6.9%	[80]
				Diarrhea	5.8%	
			926 patients without severe COVID-19 infection	Nausea and vomiting	4.6%	
				Diarrhea	3.5%	
2	138 patients	56	36 patients in ICU with critical infection 102 in non-ICU with COVID-19 infection	Diarrhea	10.1%	[92]
				Anorexia	39.9%	
				Nausea	10.1%	
				Vomiting	3.6%	
				Abdominal pain	2.2%	
3	305 patients	57	46 patients with critical infection 259 non-critical COVID-19 infection	Diarrhea	49.5%	[103]
				Loss of appetite	50.2%	
				Nausea	29.4%	
				Vomiting	15.9%	
				Abdominal pain	6.0%	
4	52 patients	59.7	All patients with severe infection of COVID-19	Gastrointestinal haemorrhage	4%	[104]
				Vomiting	4%	
5	73 patients	10 months to 78 years	All patients with COVID-19 infection	Diarrhea	35.6%	[105]
				Gastrointestinal bleeding	13.7%	
6	51 patients	49	All patients with moderate COVID-19 infection	Diarrhea	10%	[106]
				Nausea	6%	

## 8. Renal Manifestations

The renal system in another soft target of COVID-19. The damage to the kidneys is obvious, as the virus is known to bind ACE2 receptors in renal tubular cells. Therefore, post-COVID-19 kidney damages can be observed in some COVID-19 patients. Previously, a correlation of SARS-CoV-1 and MERS-CoV with renal disorders was reported, including acute renal impairment, acute kidney injury (AKI), hematuria and proteinuria [104,107]. Chu et al. (2005) studied 536 patients suffering from SARS-CoV-1 viral infection and reported that 6.7% of patients developed AKI within about 5 to 48 days after the onset of the infection. Among the 536 patients, 91.7% died, which is a significantly higher mortality rate when compared with patients suffering from SARS-CoV-1 without renal impairment [107].

If SARS-CoV-1 and SARS-CoV-2 are compared, a higher incidence of AKI was noticed in COVID-19 patients, including 0.5–29% of patients with COVID-19 in China and 37% in the USA, with 14% requiring dialysis [104,108,109]. Cheng et al. (2020) noticed that AKI affected 5.1% of the 701 COVID-19 patients [108]. The risk of mortality increased four-fold among patients with stage 3 acute kidney injury. Furthermore, Robbins-Juarez et al. (2020) performed a meta-analysis including over 13,000 patients with SARS-CoV-2 infection. The prevalence of AKI was 17% [110].

### Expected Long-Term Renal Health Effects

Studies focusing on long-term effects of COVID-19 on kidney damage are few and inconclusive. It is difficult to ascertain the possible health complications amongst these patients. However, patients with pre-existing comorbidities during infections, such as diabetes and/or other nephrological disorders, are at higher risk of developing renal impairments as long-term consequences. Therefore, patients with significant renal impairment must have mandatory annual medical follow-ups to prevent and treat further renal deterioration.

## 9. Psycho-Social Manifestations

One of the major consequences due to the COVID-19 pandemic in humans is mental health, which includes various psychosocial disorders such as depression, anxiety, panic disorder, sleep disturbance, chronic fatigue syndrome, myalgic encephalomyelitis and post-traumatic stress disorder [111,112]. These disorders are not only confined to the recovered patients, but also to the large population of the world, due to several reasons, such as the death or suffering of close individual, stress of lockdown, inability to adjust to changes brought about by pandemic and loss of job.

It has been observed that patients suffering from SARS-CoV-2 experienced long-term health effects, including widespread fatigue, psychological distress, chronic pain and disturbed sleep, which leads to failure to return productive work for at least one year after the acute trauma of pandemic [100,113]. Wu et al. (2005) first noticed that 10–18% of SARS-CoV-1-recovered patients exhibited symptoms related to depression, anxiety and post-traumatic stress disorders with elevated risk of severe symptoms of virus that have low emotional support and high-perceived life threats. Another group of scientists from Hong Kong studied a group of SARS-CoV-1-infected patients and reported that the patients experienced severe depression, pain disorders, PTSD and panic disorders in about 39, 36, 55 and 33% of patients, respectively within about 31 months to 50 months post-infection [114,115].

**Figure 5:**
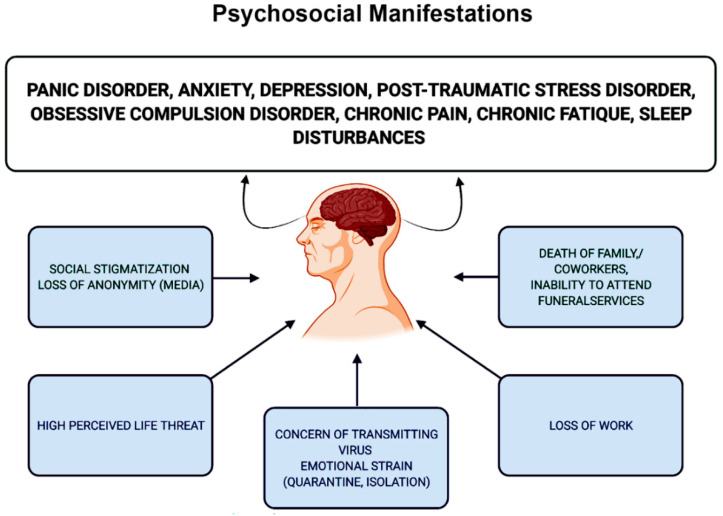
Major psychosocial manifestations in recovered patients stemming from the changes in the thought process and world brought about by the COVID-19 pandemic.

Similarly, a group of medical experts observed the psychosocial manifestations of SARS-CoV-2 on 714 hospitalized patients and they noticed that around 96% of total patients experienced a degree of post-traumatic stress disorder symptoms [116]. Studies strongly suggest that the COVID-19 pandemic could have a substantial impact on the mental health of survivors, including OCD, anxiety, chronic pain, panic disorder, PTSD and depression [116]. Moreover, coronavirus infection can also lead to chronic fatigue and sleep disturbance in patients. As shown in Figure 5, potential stressors have been classified those that can cause these psychosocial manifestations as long-term consequences of the COVID-19 pandemic, including the loss of work, death of family and loved ones, inability to attend funerals of closed ones, concern of transmitting the virus to loved ones, high perceived life threat, emotional strain from quarantine and social stigmatization. Therefore, it has been suggested that psychosocial complications are emerging as important issues in patients infected with COVID-19, which are expected to become the long-term consequences of SARS-CoV-2 infection in upcoming years in pandemic-affected people and needs to be research further by medical experts and psychologists.

### Expected Long-Term Psychosocial Health Effects

The pandemic has been prevalent for the past two years and almost everyone has been affected by the changes it has brought. It has indeed been evident that people are becoming more susceptible to anxiety and depression along with post-traumatic stress disorders and memory complaints. This is expected to have an effect on the happiness and behavior of the public around the world.

## 10. Conclusions

During this COVID-19 pandemic, we need ample research to understand the biology of the virus, particularly new variants, such as the delta and omicron, and our improved understanding of virus type(s) may help in preventing and/or minimizing long-term health consequences. The interactions between humans and virus have been known since the emergence of human beings. It has been studied that about 8% of our genome contains viral gene sequences, indicating extensive co-evolutionary relationship. In spite of this, the long-term effects of viral infections on human health remains largely under-appreciated, which leads to severe health consequences for many years after the pandemic. Although there are some studies that have analyzed the expected consequences of long COVID-19 [117,118,119,120], our study critically assessed the long-term health consequences of SARS-CoV-2 in relation with two similar viruses, namely, MERS-CoV and SARS-CoV-1. Along with it, many studies have focused on a particular region or on a single population of virus-infected patients in some hospitals. In this study, the expected future health outcome were estimated amongst SARS-CoV-2-infected patients with the long-term consequences of COVID-19 over the major systems of human body, including CNS, pulmonary, hematologic, cardiovascular, psychosocial, gastrointestinal and renal systems. Further research is warranted to understand the molecular mechanisms of COVID-19 consequences on human body systems. Additional testing and preventive measures could be implemented in clinical settings to prevent COVID-19 complications on human health.

## Figures and Tables

**Table 1:** Comparative analysis of viruses that cause differences in the epidemic scale and the severity of the consequences.

Characteristics	SARS-CoV-1	MERS-CoV	SARS-CoV-2
Virus species	Severe acute respiratory syndrome coronavirus-1	Middle east respiratory syndrome coronavirus	Severe acute respiratory syndrome coronavirus-2
First identified location	Guangdong, China	Jeddah, Saudi Arabia	Wuhan, China
Epidemic period	2002–2003	2012–ongoing	2019–present
Receptors on the human body for attachment	ACE-2	DPP4, CD-6 in respiratory epithelial cell	ACE-2
Symptoms	Fever, headache, dry cough, shortness of breath, without upper respiratory tract symptoms	Fever, cough, shortness of breath, diarrhea, pneumonia	Fever, cough, shortness of breath, loss of taste or smell, chest pain
Incubation period	2–10 days	14 days	2–14 days
Transmission	Respiratory droplet (person to person)	Respiratory droplet (person to person), non-human to human	Respiratory droplet (person to person)
T-cell immune response	Reduced total, T_c_ and T_h_ cells; long-term reaction against S and N proteins; greater frequency and quantity of CD8+ vs. CD4+	Reduced T_h2_ cells; long-term reaction against S, M, N and E proteins; greater frequency and quantity of CD8+	Reduced total, T_c_ and T_h_ cells; long-term reaction against S, N, nsp7, nsp13 and ORF1 proteins; greater frequency and quantity of CD8+ vs. CD4+
Humoral response	IgG, IgM and IgA production; detection in first the two weeks of infection	IgG, IgM and IgA production; detection in first two weeks of infection	IgG, IgM, IgA and IgE production; detection in first week of infection
Natural host	Bat	Bat	Bat
Reservoir host	Civets, cats and bats	Dromedary camels	-
Active cases	No report since 2004	Active	Active
Infections	8098	2521+	569,896,067+
Deaths	10% of infected patients, but can increase to 50% in case of age higher than 60 years	Fatality rate is 34% (every 3–4 patients out of 10, i.e., infected from MERS-CoV)	3–4 out of every 10 infected patients (outbreak in progress)
